# Dissociation and Pain-Catastrophizing: Absorptive Detachment as a Higher-Order Factor in Control of Pain-Related Fearful Anticipations Prior to Total Knee Arthroplasty (TKA)

**DOI:** 10.3390/jcm8050697

**Published:** 2019-05-16

**Authors:** Matthias Vogel, Martin Krippl, Lydia Frenzel, Christian Riediger, Jörg Frommer, Christoph Lohmann, Sebastian Illiger

**Affiliations:** 1 Universitätsklinik für Psychosomatische Medizin und Psychotherapie der Otto-von-Guericke-Universität Magdeburg, Leipziger Straße 44, 39120 Magdeburg, Germany; Lydia.frenzel@med.ovgu.de (L.F.); joerg.frommer@med.ovgu.de (J.F.); 2 Institut für Psychologie der Otto-von-Guericke-Universität Magdeburg, Universitätsplatz 2, Geb. 24, 39106 Magdeburg, Germany; martin.krippl@ovgu.de; 3 Universitätsklinik für Orthopädie der Otto-von-Guericke-Universität Magdeburg, Leipziger Straße 44, 39120 Magdeburg, Germany; christian.riediger@med.ovgu.de (C.R.); christoph.lohmann@med.ovgu.de (C.L.); sebastian.illiger@med.ovgu.de (S.I.)

**Keywords:** total knee arthroplasty (TKA), pain-catastrophizing, dissociation, hierarchical structure

## Abstract

Total Knee Arthroplasty (TKA) is the ultima-ratio therapy for knee-osteoarthritis (OA), which is a paradigmatic condition of chronic pain. A hierarchical organization may explain the reported covariation of pain-catastrophizing (PC) and dissociation, which is a trauma-related psychopathology. This study tests the hypotheses of an overlap and hierarchical organization of the two constructs, PC and dissociation, respectively, using the Western Ontario and McMaster Universities Osteoarthritis Index (WOMAC), the Childhood Trauma Screener (CTS), a shortened version of the Dissociative Experiences Scale (FDS-20), the Brief Symptom Inventory (BSI-18), the Pain-Catastrophizing Scale (PCS), and the Tampa Scale of Kinesiophobia (TSK) in 93 participants with knee-OA and TKA. Non-parametric correlation, linear regression, and an exploratory factor analysis comprising the PCS and the FDS-20 in aggregate were run. The three factors: (1) PC factor, (2) absorptive detachment, and (3) conversion altogether explained 60% of the variance of the two scales. Dissociative factors were related to childhood trauma, and the PC-factor to knee-pain. The latter was predicted by absorptive detachment, i.e., disrupted perception interfering with the integration of trauma-related experiences possibly including invasive surgery. Absorptive detachment represents negative affectivity and is in control of pain-related anxieties (including PC). The clinical associations of trauma, psychopathology, and maladaptation after TKA may be reflections of this latent hierarchical organization of trauma-related dissociation and PC.

## 1. Introduction

### 1.1. Hierarchy of Pain-Related Anxieties

The cognitive behavioral construct of pain-catastrophizing (PC) refers to “an exaggerated negative mental set brought to bear during actual or anticipated painful experience” [1]. It is known to exert a direct influence on a patient’s response to the experience of pain [2]. Moreover, being a content-specific (i.e., pain-related) construct, PC overlaps with other constructs of negative affectivity (e.g., generic constructs of anxiety, aggression, and alienation) [3]. Therefore, Vancleef et al. [3] have reinforced the notion of a hierarchical structure of pain-related negative emotional constructs [4], in which content-specific constructs directly related to pain reside in close vicinity to the actual experience of pain. On the contrary, generic constructs of (negative) affectivity function as higher-order factors in the control of content-specific anxiety. For example, a common factorization of PC and higher order negative affect, as well as coping strategies, revealed a functional hierarchy involving PC and illness focused coping [5]. Likewise, Kleiman et al. [6] corroborated the hierarchical model by means of a factor analytic study examining the factor structure of measures of negative affect and content-related anxieties, including PC in aggregate. Those authors extracted one common factor termed sensitivity to pain traumatization, which represents a pain-related stress reaction of a posttraumatic character (e.g., intrusive thoughts, avoidance, and arousal). Dissociative phenomena are entangled with negative affect as well, partly as a result of their emotionally destabilizing potential [7], partly by virtue of their negative affective nature, which led Watson and Clark [8] and Lilienfeld et al. [4] to subsume alienation (i.e., derealisation/depersonalization) under negative affect. Likewise, Tellegen [9] and Patrick and Kramer [10] conceptualized alienation (or estrangement) and absorption as components of negative affectivity. Therefore, not surprisingly, high negative affectivity is linked to dissociative phenomena in the frame of posttraumatic stress disorder (PTSD) [11]. Not least, Kleiman et al. [6] postulate symptoms of emotional numbing (i.e., pain-related emotional detachment) to be reflective of such posttraumatic symptomatology, which occurs in response to pain. Similar to the factor analytic studies in personality disorder research, those studies indicate the possibility of explaining the covariation among the observed pain-related variables through the use of latent constructs, brought to light by factor analysis [12], also with regards to research on chronic pain and its nested psychological correlates. The knowledge of such systematic interactions between PC, which is an important predictor of postoperative pain [13], and posttraumatic symptomatology likely offers hints on adequate psychotherapeutic strategies, as well. For example, dissociative symptoms, if proven to contribute to chronic pain, would call for a trauma-specific therapy also in this context.

### 1.2. Dissociation and Pain-Catastrophizing

The DSM-5 defines dissociative symptomatology as the disruption of and /or discontinuity in the normal integration of consciousness, memory, identity, emotion, perception, body representation, motor control, and behavior [14]. Dissociation has been hypothesized as a survival mechanism for surviving a trauma (e.g., sexual) [15]. Holmes et al. [16] have suggested distinct classes of dissociative symptoms, namely detachment–dissociation and compartmentalization–dissociation, at which the former is characterized by a sense of separation from the body, self, and environment, whereas the latter reflects the temporary loss of deliberate control over distinct systems, e.g., memory [17]. Regarding chronic pain, dissociation is believed to serve to minimize memories of traumatic events, including the minimization or magnification of the pain perception (the pain focus) [18]. Dissociative symptoms are frequent in trauma-related psychiatric disorders [19], and, in addition, also occur in pain-prone medical conditions, such as Osteoarthritis (OA) and rheumatoid arthritis [20]. Likewise, PC is linked to chronic pain and to worse outcomes of OA-related arthroplasty [21]. Notably, preoperative PC increases pain perception by means of magnifying pain focus [22], and preoperative pain, in turn, is a strong predictor of the postoperative algofunction (AF) [23], i.e., the combined status regarding knee-pain and knee function. 

### 1.3. Psychosomatic Aspects of Osteoarthritis and Total Knee Arthroplasty

OA is a degenerative joint disease causing pain and stiffness, is among the leading causes of chronic pain and disability [24], and serves well as an example of the progressive, complex, and multifaceted nature of chronic pain [25]. TKA represents the ultimate therapeutic option for OA of the knee. However, about a quarter of the patients undergoing TKA experience neither pain-relief nor functional restoration after TKA without there being any detectable medical cause [26]. Moreover, OA is known to coincide with depression, anxiety, and PC, with exactly these psychological circumstances signifying greater pain even after TKA [27]. Those reports correspond well to the prediction of postoperative outcomes by personality characteristics, such as neuroticism or borderline personality [13,28]. Moreover, chronic pain is connected to specific anxieties related to pain, which include kinesiophobia in addition to PC. The fear avoidance model of chronic pain posits a circular interaction between the pain focus (that is PC), the perception of pain as well as the fear of movement and re-injury. As a result, the individual is prompted to withdraw from activity and social surroundings to the effect of reduced participation, a less healthy lifestyle, and worsened pain [28]. 

### 1.4. Linking Pain-Catastrophizing and Dissociation

Experimental research [29] suggests an overlap between PC and dissociation, and theoretical considerations [30] do so regarding catastrophic cognitions and dissociative experiences. This assumption is based on the notion that symptoms, be it dissociation or chronic pain, are perceived and processed in accordance to the meaning which the individual ascribes to them. Specifically, according to Ehlers and Steil [30], the co-occurrence of helplessness and detachment dissociation serves to maintain mental control by preventing exposure to feared material. This theorizing is consistent with the results of Gómez-Pérez et al. [29], who demonstrated the simultaneous elicitation of PC and dissociative experiences through a cold pressor task, thus suggesting each of the idiosyncratic meanings of those symptoms to be mutually influenced. Given the relationship of PC and dissociation with chronic pain, both likely contribute to the adaptation to chronic pain, possibly based on an underlying hierarchical structure [3]. Interestingly, researchers have found PC and chronic pain to be linked to childhood abuse [31], paralleling the respective associations between dissociation and prior trauma [32].

Considering the relationship between PC and childhood trauma, on the one hand, and between dissociation and chronic pain on the other, let alone with the hierarchical model in mind, the question arises as to how those symptoms are connected. Lynn et al. [33] and Giesbrecht et al. [34] have proposed the action of cognitive processes in dissociation, which may find its reflection in a clinical population treated for chronic pain in the form of an interaction and functional hierarchy between the two constructs [3]. The present study tests the hypothesis of an overlap between dissociation and PC in patients with end-stage knee-OA and chronic pain scheduled for TKA, who are known to be put under an enormous stress by the imminence of knee-surgery, hypothesizing an overlap which, in addition, is structured by distinguishable categories of dissociation interacting with the respective categories of pain-catastrophizing as predicted by the hierarchical model of pain-related negative affective constructs [4]. We therefore investigate the hierarchically structured organization possibly underlying PC and dissociation in people with chronic pain scheduled for TKA by means of an exploratory factor analysis. This is based on the assumption that dissociation would function equivalently to higher-order constructs of negative affect. Moreover, we assume pain-related anxieties, including PC and kinesiophobia, to be hierarchically nested, thus possibly lending an explanation to the covariation of the two constructs by the identification of a latent construct [12]. The rationale behind this procedure is to improve our theoretical understanding of psychological dispositions, including childhood trauma and its (adult) correlate, dissociation, for the chronicity of TKA-related pain. 

## 2. Materials and Methods

All procedures performed in this study were in accordance with the ethical standards of the institutional and/or national research committee and with the 1964 Helsinki declaration and its later amendments or comparable ethical standards. Informed consent was obtained from all individual participants included in the study. 

### 2.1. Sample 

A previous study [35] reported a moderate correlation (r = 0.32) between the Dissociative Experiences Scale [36] and the Cognitive Failure Questionnaire [37]. Given that the PC is also reflecting a cognitive construct, we used this finding as the basis of our power calculation, which we conducted by means of G*power (Version 3.1.9.2) [38], arriving at the minimum sample size of *n* = 74 (two-tailed, power = 80). 

However, 98 patients (53% female) with an average age of 64.64 (±10.55) years (males: 64.27 ± 9.87, females: 64.98 ± 11.22) scheduled for elective primary TKA for OA were consecutively included in the present study. The study was approved by the local Institutional Review Board. Inclusion was based on the aspect of primary TKA for OA and age > 18, while exclusion pertained to the presence of records of major psychiatric illness. The assessment took place only one to two days before the operation for the sake of which participants were admitted to the orthopedic department. We enrolled the participants of the present study consecutively between 2015 and 2017.

### 2.2. Questionnaire Measures

Knee pain and function were assessed using the Western Ontario and McMaster Universities Osteoarthritis Index (WOMAC) pain and function subscales (WOMAC A and WOMAC C). Cronbachs α’s of the WOMAC range from 0.8 to 0.96 and its psychometric properties are judged as being good [39]. The WOMAC used in this study was the Likert version in the format of a numerical rating scale ranging from 0 to 10. 

The brief symptom inventory (BSI-18), a short version of the Symptom Check List 90, assesses symptoms of depression, anxiety, and somatization in three subscales. Internal consistency for the subscales ranges between 0.79 and 0.91, discriminant and convergent validity are deemed good, and the scale is useful as a screening for psychological distress in physically ill populations [40]. This distress is particularly marked prior to TKA and is also a predictor of a worse postoperative algofunction.

The Fragebogen zu dissoziativen Symptomen (FDS) [41] represents the German version of the Dissociative Experiences Scale, of which we used the short form (FDS-20). The FDS-20 is composed of the most sensitive items of the longer version on the condition they reach a Cronbachs α of at least 0.9. The total scale has a good internal consistency (α = 0.93). Items are rated in terms of frequency on a scale ranging from never present (0%) to always present (100%). However, for the purpose of the exploratory factor analysis, we transformed the data into four categories reflecting the following ranges of values: 0%–25%, 26%–50%, 51%–75%, and 76%–100% (of the time), respectively. Rodewald et al. [42] determined the cut-off of the FDS-20 to be 13, allowing for differentiation between severe and non-severe dissociation. Means of the FDS-20 are reported to range from 5.0 in non-psychiatric samples to 25.43 in personality disorders [41]. The factorial structure of the Dissociative Experiences Scale does not apply to its short version, FDS-20.

The Pain-Catastrophizing Scale (PCS) is a 13-item rating-scale comprising the subscales rumination (PCS-Rumi), magnification (PCS-Magni), and helplessness (PCS-Help) [43]. It assesses thoughts and feelings about the pain experience on a 5 point Likert scale. The PCS has proven adequate to excellent internal consistency (Cronbachs α’s: total score: 0.87, PCS-Rumi: 0.87, PCS-Magni: 0.66, PCS-Help: 0.78). The cut-off serving the distinction between severe and non-severe pain-catastrophizing was set at 30.

The Tampa Scale of Kinesiophobia (TSK) is a 13 item rating scale rated on a 4 point Likert scale. Assessing fear of movement and re-injury, it is a valid and reliable instrument with the Cronbach’s α being 0.73 for its German version [44]. The TSK is divided into two subscales termed activity avoidance (AA) and somatic focus (SF). Kinesiophobia is essential to fear avoidance, which makes it a relevant construct also for pain-related psycho-traumatology.

The CTS is a five-item-self-report instrument assessing childhood emotional, physical, and sexual abuse, as well as childhood emotional and physical neglect. The CTS is derived from the Childhood Trauma Questionnaire for use as a screening for childhood trauma. Cronbach’s α of the CTS is 0.75, and it is judged to be reliable and valid for its purpose [45]. 

### 2.3. Statistical Analysis

We used t-testing to compare groups (e.g., male/female) and non-parametric correlations (Kendall’s Tau) to explore the relationships between continuous variables. The latter choice corresponds to the skewed distribution of dissociative symptoms in non-psychiatric populations [41]. Principal axis factor analysis (PAF) [46] was applied for the 33 items of the FDS-20 and the PCS. We were advised to do so, because the factorial structure of the Dissociative Experiences Scale does not apply to its short version and, in addition, this chosen procedure suits our intention to identify the presumed latent structure of the two scales and their observed covariation [4]. The factors were based on the Kaiser-Guttman-Criterion (eigenvalue > 1) [47] and rotated using the promax method. After that, parallel analysis [48] was applied, which revealed three factors to extract. We discarded those items, which had their highest loading in the rotated loading matrices on a factor other than 1, 2, or 3. 

The factor scores represent the criteria used in the linear regression analyses. We selected the corresponding independent variables according to their significance in the preceding non-parametric correlations. All regressions were controlled for age, gender, and the severity of dissociation or PC (according to the respective cut-off scores), respectively, each depending on their use as a predictor. 

## 3. Results

### 3.1. Description of the Sample

The sample was comprised of 98 participants (53% female) with an average age of 64.64 (±10.55) years (males: 64.27 ± 9.87, females: 64.98 ± 11.22). Five patients refused to participate due to reluctance to fill in questionnaires. In case of missing data, the participants were contacted and the values recovered on the occasion of follow-up visits in the orthopedic university clinic. Table 1 displays the sociodemographic description of the sample with complete data (*n* = 93). The mean score (SD) and ranges of the WOMAC A and C scales were as follows: A = 5.3 (2.05) and 0.2 to 10.0, C = 4.88 (2.3) and 0.29 to 12.53.

Hence, the FDS-20 scores were skewed with most participants reporting only low levels of dissociation as expected in a non-psychiatric sample [41]. In total, 22 participants (23.66%) endorsed dissociation (FDS-total score) at 0% of the time. The mean (SD) of the TSK and the BSI were 21.09 (6.24) and 6.59 (7.14). The mean (SD) scores of the CTS-subscales were: EN: 1.70 (0.93); PA: 1.47 (0.9); EA: 1.32 (0.8); SA: 1.1 (0.39); and PN: 2.23 (1.27). There were significant (Pearson) correlations between the TSK and PCS (r = 0.35, *p* < 0.01), the GSI and pain-related anxieties (TSK: r = 0.21, *p* < 0.01; PCS: r = 0.43, *p* < 0.01), and between the FDS and the TSK (r = 0.22, *p* < 0.01). Table 2 shows the specific correlational matrix between the PCS and the FDS-20, respectively. 

### 3.2. Common Factorization of the PCS and FDS-20:

Kaiser-Meyer-Olkin-statistics (KMO) for testing the usability of data for exploratory factor analysis revealed a very good result (0.901). Parallel analysis after PAF-analysis and promax-rotation revealed three factors. 

Table 3 shows the latent factors at which the exploratory factor analysis arrived, the respective proportion of explained variance, and the wording and the mapping on the original scale. Items with the highest loadings on a factor represent that factor (bold face in Table 3). The loss of 13 (factors 4 to 7) of the 33 items, with which the PAF was fed, is due to the determination of three factors by parallel analysis. An item was allocated to a certain factor, on the condition that its highest loading was on that factor. The lowest loading on a factor was 0.38 (Table 3). The most influential factor reflects a compound of the subscales of the PCS, apparently reflecting the core affective and cognitive responses to pain typically displayed by patients with chronic pain. It is therefore referred to as the pain-catastrophizing factor (PC-factor). The second factor, termed absorptive detachment in reference to Allen, Console, and Lewis (49), contains items that map onto the subscales of amnesia and derealisation of the FDS. The third factor reflects the conversion subscale of the FDS and was named accordingly. Mean (SD) of the factor scores for low/high dissociators (in accordance to the respective cut-off point) were the following: Helplessness: −0.12 (0.9)/1.59 (0.71); absorptive detachment: −0.23 (0.32)/2.87 (2.15); conversion: −0.2 (0.62)/1.98 (1.81).

### 3.3. Factor Correlations and Interrelation

Table 4 shows the correlational matrix of these factors as well as their correlations with the clinical variables of interest. The PC-factor and the absorptive detachment factor were highly correlated with each other (cf. Table 4), but not with the factor conversion. The event of an item loading highly on two factors did not occur among factors 1 to 3, although the three items of factor 6 had their second highest loadings on factor 1. Regarding the BSI-18, the conversion factor was only linked to somatization, whereas the other two were linked to depression and anxiety, as well. The conversion factor—unlike its companions—lacked an association with TSK-subscales. Knee-pain and function were related to the PC-factor only, whereas childhood trauma solely showed correlations with dissociative factors. Based on the cut-off score of the FDS-20, six participants qualified as severe dissociators.

Table 5 displays the results of the stepwise linear regression analyses, with the dependent variables being the factors 1, 2 and 3, and the predictors having been chosen based on the finding of a significant non-parametric correlation with the dependent variable in the previous step. The PC-factor was best predicted by the absorptive detachment-factor, but also by depression. The former was best predicted by activity avoidance, and the conversion factor by high PC. Hence, the two scales were separated by the factor analysis, but regression revealed their specific interactions in patients with imminent TKA. 

## 4. Discussion

The present study explored the relationships between childhood trauma, PC, and dissociation in patients with end-stage knee osteoarthritis scheduled for TKA, based on the assumption of (1) a phenomenological overlap and clinical interaction [29], and (2) a hierarchical organization [3] between dissociation and PC [33]. Pain-related anxieties and negative affect (as represented by the global severity index of the BSI-18) proved highly intercorrelated, as did the FDS and pain-related anxieties, suggesting that negative affect, pain-related anxieties, and dissociation are nested. This prerequisite of the hierarchical model of pain-related anxieties aside, the factor analysis separated the FDS and the PCS, yielding one PCS-(catastrophizing) and two FDS-(dissociative) factors. The three factors together explained about 60% of the variance of the two scales and were subsequently correlated to knee-pain and function, as well as to psychopathological distress, including kinesiophobia, and to childhood trauma scores. All factors showed correlations with psychopathological distress and kinesiophobia, but the latter was more closely related to the PC-factor. Finally, linear regression showed that the latter (accounting for 44% of the variance of the two scales according to the factor analysis) was best predicted by the absorptive detachment factor. Contrarily, the conversion factor was predicted by high PC, and the absorptive detachment factor by activity avoidance. Thus, the factors apparently differ with regard to their elicitation as well as to their maintenance in patients with chronic pain undergoing TKA. Interestingly, the correlational patterns reveal specific associations between PC and knee-pain and function, and between dissociation and childhood trauma, respectively. On the contrary, all three factors showed close relationships with psychopathological distress, except for the conversion factor, which was not linked to kinesiophobia. Importantly, the regression analyses controlled for severe dissociation and severe PC, respectively, based on cut-offs, and the correlations were non-parametric, hence the present results do take the skewed distribution of dissociative symptoms in this non-psychiatric population into account. 

### 4.1. A Topography of Specific Associations

The absorptive detachment factor is made up by a choice of dissociative symptoms, representing derealisation/depersonalisation (also referred to as detachment–dissociation) [15] on the one hand, and items, that are best described as relating to mal-integrated memory (amnesia) and the corresponding disruptions of identity, on the other. The latter result from the failure to properly contextualize and encode personal and (auto-) biographic memories constitutive of the self [49,50]. These specific dissociative experiences represent severe dissociation, as they are causing interruptions to the stream of consciousness, leading to gaps in identity and to ruminating, self-absorbed states (absorption) due to the preoccupation with, e.g., dissociative hallucinations [51] or other forms of compartmentalized traumatic memories. Likewise, jamais-vu (i.e., spurious non-familiarity) experiences are epiphenomena of severe dissociation [52], which could explain their high loading on the absorptive detachment-factor. Thus, this latent factor could be indicative of a dynamic interaction between states of detachment from the body, self, and/or environment, on the one hand, and the resulting lapses in memory formation, on the other. Moreover, the pain-specific construct of pain-related helplessness behaved as predicted by the hierarchical model and appears to be hierarchically nested within the generic (higher-order) construct of negative affect, which, as the present results suggest, does include detachment-dissociation. 

### 4.2. Dissociative Coping: Legacy of Childhood Trauma in TKA and Beyond

This interaction is likely linear, dependent on the severity of dissociative symptoms and therefore possibly driven by the severe dissociators in the present study. Nevertheless, our results suggest a principal covariation of the two constructs, and are therefore in-line with [3], who incorporated alienation (i.e., detachment dissociation) in their concept of pain-related negative affect, although those authors do not lay their focus on dissociation. Comparable, specific interactions between the subtypes of dissociation, compartmentalization–dissociation, and detachment–dissociation, and different kinds of psychopathology, as well as their relation to childhood trauma have been described regarding psychiatric patients [53]. The respective differences pertain to the differential influence of detachment–dissociation and compartmentalization–dissociation, respectively, on the process of coping with psychic, and possibly also psychosomatic, as well as physical distress. While the population under study here is not psychiatric, it is facing TKA instantaneously and therefore under an enormous psychological strain. The latter includes a magnifying pain focus, which may initiate a dissociative response [29], possibly following a similar pattern as has been revealed in a psychiatric population, in principle. Dissociation is viewed as a means of coping with adversities, such as mental or physical harm, which makes it relevant to coping with, e.g., severe psychopathology or major surgery [54]. The latter may be viewed as an interpersonal act of violating the bodily integrity, especially based on the prior experience of interpersonal trauma.

Unlike other authors [30], we did not find an association between childhood trauma and PC. Rather, the present findings could suggest that those reports reflect the extent to which variances are being shared between measures of PC and dissociation. Accordingly, the type of trauma seemingly involved in the dynamics between PC and dissociation for the most part in the present study is childhood abuse, which Sansone et al. [30] also found to be linked to PC. Hence, the present findings suggest preoperative PC not only to be embedded in psychopathological distress, but also to be hierarchically nested within the more content-general construct of detachment–dissociation. Considering the trauma model of dissociation, which posits the causation of dissociative experiences by trauma [32], any cue reminiscent of an experienced trauma could therefore sensitize the individual by creating the expectation of another aggressive and hurtful encounter happening [30]. Regarding the dissociative factors, their prediction by activity avoidance and high pain catastrophizing surprises in that detachment was linked to kinesiophobia (or more precisely, activity avoidance). Kinesiophobia, apart from being correlated with catastrophizing, is obviously linked to self-absorbing inner experience and phantasies. That state possibly prevents the individual from being occupied with any activity other than that. Moreover, the link between the conversion factor and high PC underscores the relevance of phobic mechanisms for coping with major surgery [55]. Accordingly, joint function after TKA has been related to anxiety levels [56], a finding which possibly points to a subgroup of patients undergoing TKA, who are also suffering from trauma and dissociation.

Hence, in-line with the experimental data [29] indicating a dynamic relationship between dissociation and PC, the present results suggest dissociation severe enough to interfere with the consolidation of one’s identity to maximize the psychopathological distress in traumatized individuals with TKA. This process seems to be driven by a lack of inner cohesion (that is, detachment from certain aspects of one’s body, self, or environment) and by the systematic interactions between PC and trauma-related psychopathology. Accordingly, helplessness has been found to mediate the effect of catastrophizing on pain [57]. Inner cohesion is a reasonable prerequisite of effective self-regulation and competent mastery; consequently, unfavorable coping styles, such as rumination and self-blame, have been linked to detachment, a finding [58] possibly relevant to somato-psychic pathology, on a larger scale.

### 4.3. Synergism Between Pain-Catastrophizing and Dissociation

This notwithstanding, another effective way to induce dissociative symptoms, as well as PC in patients with TKA, apparently is to develop emotional distress, which—as many studies could demonstrate [26,28]—is especially pronounced in the face of imminent TKA. While affection with feelings of anxiety and depression may be a frequent reaction to facing invasive surgery for most people [59], the present study illustrates that it echoes the hierarchically structured interaction of detachment–dissociation and PC. As to the correlational conflation of the FDS-20 and the PCS in this study, it is consistent with a synergism rather than with the assumption of an antagonistic nature of their relationship [60]. This is also evident from studies reporting that the two forms of psychopathology converge to the effect of increasing the propensity for nocebo-like reactions [61], as one could call the events of impaired postoperative algofunction after TKA in the absence of medical causes. Moreover, from a theoretical point of view, the universal nature of the non-parametric correlation between the FDS-scale and the PCS, as well as the prediction of pain-related helplessness by detachment–dissociation correspond to the literature that conceives dissociation as being bound to cognitive failures. This stance [34], however, does not necessarily involve a functional hierarchy. Nevertheless, the latter could reconcile the controversy on the nature of dissociation at least in relation to pain-related cognitions. Moreover, the present results imply an overlap of PC, as assessed by the PCS, with various kinds of dissociative symptomatology.

### 4.4. Forced to the Knee: The Special Implications of Knee-Osteoarthritis

In accordance to this, several studies reported an association of PC either with amnesia or with depersonalization/derealisation, or both [29,62]. However, the cited studies are concerned with different samples and, most of all, different pain sites. In order to understand the psychological strain on patients awaiting TKA, the essentiality of the knee for our two-legged mobility ought to be considered: The respective patients with TKA are known to suffer markedly from functional problems as a result of malposition due to fear of pain and re-injury [63], to depend on help with their personal care and routine needs [64], and to experience a faster decline in gait speed, allowing for less participation to be reached as the disorder progresses compared to OA of the hip [65]. In conclusion, the present study, albeit retrospective and cross-sectional in nature, does encourage important clinical conclusions. A subgroup of patients with TKA may have specific difficulty coping with TKA as a result of childhood trauma and dissociative symptomatology and should therefore be offered psychosocial support based on psychological screenings. Moreover, the remarkable extent of psychopathological distress born by patients with imminent TKA may lead orthopedic surgeons to administer antidepressants peri-operatively in order to counterbalance the impact of psychopathological distress on the outcomes of TKA. As to the caveats of this study, the lack of psychiatric diagnostic interviews, which can hardly be implemented in an orthopedic setting, deserves mentioning. Moreover, the sample was limited in size and restricted to patients with OA scheduled for primary TKA and may therefore not be representative of other indications. Also, the five drop-outs in this study limit the interpretability of the results, especially since we cannot describe them due to a lack of sociodemographic information, which they did not provide. Furthermore, the common factorization deployed in the present work is a controversial procedure, although it offers the possibility of explaining the observed covariation of the variables under study by the identification of unobserved, latent factors [4] and is, in addition, a proven method in personality psychology [12]. However, since 13 items were excluded from the analysis by the parallel analysis, their covariation and latent contribution to the presumed hierarchical organization of pain-related anxieties and trauma-related symptoms remains obscure, although the contribution of factors 4 to 7 to the total variance of the two scales was comparatively small. The alternative price to pay would have been a reduced factor variance in connection with a heightened error variance as a result of a forceful allocation of the 13 omitted items on factors 1 to 3 based on secondary loadings. The latter procedure would have caused error correlations to occur, which would oppose the classical test theory [66]. Nevertheless, the present study is the first to suggest absorptive detachment as a latent factor crucial for the maladaptation to pain and its chronification. Moreover, the results reinforce the notion that childhood trauma and dissociation are relevant to coping with surgery and also deserve diagnostic attention in a surgical setting.

## Figures and Tables

**Table 1:** Sociodemographic description of the sample.

Variable	N	%
**Marital Status**		
single	5	5.4
married/in a relationship	61	65.6
divorced	10	10.8
widow	15	16.1
other	2	2.2
**School**		
no school degree	2	2.15
special school	2	2.15
8 classes	25	26.88
10 classes	43	46.24
12 classes	17	17.20
other	4	4.30
**Education**		
no profess-ion/untrained	24	25.81
completed apprenticeship	46	49.46
university	16	17.2
other	7	7.53
**Accommodation**		
own home	87	93.54
other	6	6.45
**Occupation**		
full-time	28	30.11
part time	8	8.60
at home (housewife)	4	4.30
jobless	1	1.08
pension	51	54.84
other	1	1.08

Mean (SD) of the FDS and the PCS total scores were 4.66 (7.94) and 17.75 (12.35), respectively.

**Table 2:** Correlational matrix (Kendall’s tau, *p*) between the Fragebogen zu dissoziativen Symptomern-20 (FDS-20) total score and the Pain Catastrophizing Scale (PCS) total and subscale scores.

	PCS-Rumination	PCS-Magnification	PCS Helplessness	PCS-Total
FDS-20 total score	0.44 <0.01	0.36 <0.01	0.47 <0.01	0.45 <0.01

**Table 3:** Latent factors (F1-F7) of the PCS and the FDS-20, respectively, along with the respective factor loadings and wordings of the items. * The number of factors determined to be kept by parallel analysis was 3. Items with the highest loadings on a factor represent that factor (bold face).

	Factors Kept * (% Explained Variance)			Factors Lost * (% Explained Variance)				
Scale (Item nr.)	F1 (44.7)	F2 (13.25)	F3 (5.41)	F4 (3.6)	F5 (3.45)	F6 (3.23)	F7 (3.1)	Wording
**PCS (2)**	**0.94**						−0.13	Feeling one can’t go on
**PCS (1)**	**0.92**		−0.18		−0.12			Worrying all the time about whether the pain will end
**PCS (4)**	**0.92**		0.25			−0.16	−0.10	Feeling overwhelmed
**PCS (6)**	**0.86**		−0.20	0.14				Being afraid of pain getting worse
**PCS (3)**	**0.85**		0.15			−0.10		Thinking it’s never getting better
**PCS (5)**	**0.84**							Feeling one can’t stand it anymore
**PCS (8)**	**0.70**		−0.17			−0.11	0.30	Anxiously wanting pain to go away
**PCS (11)**	**0.64**		−0.18		0.15		0.32	Badly wanting pain to go away
**PCS (13)**	**0.51**	0.12			−0.15	0.22	0.35	I wonder whether something serious might happen.
**PCS (7)**	**0.44**		0.25	0.17			0.19	Thinking of other painful events
**FDS (19)**		**0.89**			0.14			Being in a familiar place but finding it unfamiliar
**FDS (20)**		**0.86**	−0.18			0.19		Feeling as though one were different people
**FDS (8)**		**0.78**	0.30	0.12		−0.15		Hearing voices inside one’s head
**FDS (13)**	−0.11	**0.59**		0.18	0.29		0.11	Other people and objects do not seem real
**FDS (15)**	0.16	**0.52**	0.21	−0.11	0.31			Abidance without movement, communication or reaction
**FDS (4)**		**0.38**	0.10	0.32	−0.23	0.33		Seeing oneself as if looking at another person
**FDS (10)**			**0.87**				0.10	Feeling one’s extremities are weak, not being able to use them
**FDS (9)**			**0.68**	0.14				Paresthesia or not feeling parts of one’s body
**FDS (18)**		0.33	**0.55**			0.16	0.12	Feeling uncertain when walking or standing
**FDS (12)**		0.39	**0.46**	−0.33	0.14	0.25		Unable to coordinate movements
**FDS (2)**		−0.16		**0.74**	0.26			Looking at the world through a fog
**FDS (3)**			0.13	**0.70**		0.12		Not recognizing one’s reflection in a mirror
**FDS (1)**	0.15	0.15		**0.51**	0.20	−0.26		Feeling one’s body is not one’s own
**FDS (16)**	0.15	0.33	−0.11	**0.43**		0.13	−0.16	Remembering past so vividly one seems to be reliving it
**FDS (17)**					**0.68**	0.22		Staring into space
**FDS (7)**		0.23		0.13	**0.64**			So involved in phantasy that it seems real
**FDS (11)**				0.22	**0.50**	0.31	−0.11	Being accused of lying when telling the truth
**FDS (6)**	−0.11		0.20			**0.68**		Finding evidence of having done things one can’t remember doing
**FDS (5)**		−0.16	0.11	0.29	0.26	**0.57**		Not sure if remembered event happened or was a dream
**FDS (14)**					0.38	**0.55**		Not sure whether one has done something or only thought about it
**PCS (9)**	0.49						**0.60**	Unable to stop thinking about pain
**PCS (10)**	0.48						**0.58**	Permanently thinking of how one wished pains to end
**PCS (12)**	0.29	−0.14	0.23			−0.15	**0.54**	Nothing one can do to relieve the pain

**Table 4:** Correlations (Kendall’s Tau, first rows and *p* values, second rows) of the factors 1 to 3 with WOMAC and psychometric scores and interfactor-correlations. WOMAC A: knee-pain; WOMAC C: knee-function; TSK: Tampa scale of kinesiophobia; CEA: childhood emotional abuse; CPA: childhood physical abuse; CSA: childhood sexual abuse; CEN: childhood emotional neglect; CPN: childhood physical neglect; GSI: global severity index, -: no correlation could be computed.

	PC-Factor	Absorptive Detachment-Factor	Conversion-Factor
**PC-factor**	-	0.36 0.000	0.09 0.27
**Absorptive detachment-factor**	0.36 0.000	-	0.02 0.76
**Conversion-factor**	0.09 0.27	0.02 0.76	-
CEN	0.06 0.50	0.21 0.02	0.13 0.15
CPA	0.15 0.09	0.20 0.03	0.20 0.03
CEA	0.18 0.05	0.30 0.001	0.10 0.29
CSA	0.13 0.16	0.07 0.44	0.06 0.55
CPN	0.10 0.2	0.22 0.009	0.15 0.08
**WOMAC A**	0.28 0.001	0.12 0.1	0.02 0.8
**WOMAC C**	0.2 0.01	0.09 0.3	−0.03 0.7
**TSK total score**	0.36 0.000	0.31 0.000	0.09 0.23
-Somatic focus	0.33 0.000	0.28 0.001	0.07 0.38
-Activity avoidance	0.32 0.000	0.31 0.000	0.13 0.10
**BSI-GSI**	0.46 0.000	0.41 0.000	0.15 0.06
-Somatisation	0.4 0.000	0.34 0.000	0.27 0.002
-Depression	0.46 0.000	0.35 0.000	0.10 0.22
-Anxiety	0.41 0.000	0.41 0.000	0.11 0.19

**Table 5:** Linear regression analyses, dependent variables: factors PC-factor, absorptive detachment, and conversion. WOMAC A/C: knee-pain/-function; CEA: childhood emotional abuse; CPA: childhood physical abuse, CEN: childhood emotional neglect, CPN: childhood physical neglect, TSK-SF: subscale somatic focus (Tampa scale of kinesiophobia), TSK-AA: subscale activity avoidance (Tampa scale of kinesiophobia), BSI: brief symptom inventory, GSI: general severity index (summary score of the BSI).

Dependent Variable	Predictor	B	SE	β	t	p	CI Lower	CI Upper
PC-factor (total model: df = 12; F = 7.03; *p* = 0.000 R^2^ = 0.61)	Gender	0.18	0.20	0.09	0.94	0.35	−0.21	0.58
	Age	−0.26	0.01	−0.26	−2.57	0.01	−0.05	0.01
	CEA	0.03	0.16	0.02	0.18	0.86	−0.30	0.36
	Absorptive detachment	0.35	0.15	0.38	2.37	0.02	0.05	0.65
	WomacA	0.05	0.08	0.10	0.61	0.55	−0.12	0.22
	WomacC	0.02	0.08	0.04	0.27	0.79	−0.14	0.18
	TSK-SF	0.02	0.05	0.05	0.40	0.69	−0.07	0.11
	TSK-AA	0.01	0.04	0.05	0.38	0.71	−0.06	0.09
	BSI-Somatisation	0.12	0.09	0.26	1.41	0.17	−0.05	0.29
	BSI-Depression	0.14	0.06	0.322	2.13	0.04	0.008	0.26
	BSI-anxiety	−0.01	−0.07	−0.01	−0.08	0.94	−0.14	0.13
	High dissociation	−0.93	0.64	−0.26	−1.45	0.15	−2.22	0.36
Factor absorptive detachment (df = 13; F = 3.25; *p* = 0.001 R^2^ = 0.41)	Gender	0.28	0.24	0.13	1.14	0.26	−0.21	0.76
	Age	0.01	0.01	0.05	0.47	0.64	−0.02	0.03
	High pain-catastrophizing	0.57	0.50	0.22	1.13	0.26	−0.43	1.56
	CPA	−0.05	0.18	−0.04	−0.27	0.79	−0.40	0.31
	CEA	0.32	0.21	0.23	1.51	0.14	−0.10	0.74
	CEN	−0.26	0.20	−0.21	−1.31	0.19	−0.65	0.14
	CPN	0.21	0.11	0.24	1.93	0.06	−0.01	0.43
	PC-factor	0.06	0.23	0.06	0.28	0.78	−0.39	0.52
	TSK-SF	−0.06	0.06	−0.17	−1.16	0.25	−0.17	0.05
	TSK-AA	0.09	0.04	0.33	2.15	0.04	0.01	0.18
	BSI-Somatisation	0.10	0.10	−0.20	−0.96	0.34	−0.10	0.30
	BSI-depression	−0.09	0.08	−0.21	−1.19	0.24	−0.25	0.06
	BSI-anxiety	0.12	0.09	0.24	1.23	0.22	−0.07	0.30
conversion factor (df = 6; F = 7.09; *p* = 0.000 R^2^ = 0.39)	Gender	0.18	0.19	0.09	0.92	0.36	−0.21	0.56
	Age	0.008	0.01	0.08	0.82	0.42	−0.01	0.03
	High pain-catastrophizing	0.79	0.28	0.33	2.78	0.007	0.22	1.35
	CPA	−0.012	0.11	−0.01	-0.10	0.92	−0.24	0.22
	GSI	−0.018	0.04	−0.12	−0.44	0.66	−0.10	0.06
	BSI-Somatisation	0.21	0.12	0.46	1.76	0.08	−0.03	0.44

B = unstandardized regression weights of predictors, SE = Standard errors of unstandardized regression weights, β = standardized regression weights, t = t-value of regression weights, *p* = *p*-value of regression weights, CI-lower = 95% confidence interval lower border of B, CI-upper = 95% confidence interval upper border of B.
